# Differentiation Therapy Targeting the β-Catenin/CBP Interaction in Pancreatic Cancer

**DOI:** 10.3390/cancers10040095

**Published:** 2018-03-29

**Authors:** Philipp Manegold, Keane K. Y. Lai, Yongfeng Wu, Jia-Ling Teo, Heinz-Josef Lenz, Yuri S. Genyk, Stephen J. Pandol, Kaijin Wu, David P. Lin, Yibu Chen, Cu Nguyen, Yi Zhao, Michael Kahn

**Affiliations:** 1 Norris Comprehensive Cancer Center, University of Southern California, Los Angeles, CA 90033, USA; philipp.manegold@uniklinik-freiburg.de (P.M.); klai@coh.org (K.K.Y.L.); wuyf001@163.com (Y.W.); jteo@coh.org (J.-L.T.); LENZ@med.usc.edu (H.-J.L.); kaijinwu@usc.edu (K.W.); cunguyen@coh.org (C.N.); yizhao@usc.edu (Y.Z.); 2 Center for Molecular Pathways and Drug Discovery, University of Southern California, Los Angeles, CA 90033, USA; 3 Department of Pathology, City of Hope National Medical Center, Duarte, CA 91010, USA; 4 Department of Molecular Medicine, Beckman Research Institute of City of Hope, Duarte, CA 91010, USA; dplin@hotmail.com; 5 City of Hope Comprehensive Cancer Center, Duarte, CA 91010, USA; 6 Southern California Research Center for Alcoholic Liver and Pancreatic Diseases and Cirrhosis, Department of Pathology, University of Southern California, Los Angeles, CA 90033, USA; Stephen.Pandol@cshs.org; 7 Department of Medicine, University of Southern California, Los Angeles, CA 90033, USA; 8 Department of Surgery, University of Southern California, Los Angeles, CA 90033, USA; yuri.genyk@health.usc.edu; 9 Pancreatic Research Program, Cedars-Sinai Medical Center, Veterans Affairs Greater Los Angeles Healthcare System, and Department of Medicine, University of California, Los Angeles, CA 90048, USA; 10 Health Sciences Libraries, University of Southern California, Los Angeles, CA 90033, USA; yibuchen@usc.edu; 11 Department of Biochemistry and Molecular Medicine, University of Southern California, Los Angeles, CA 90033, USA; 12 Department of Molecular Pharmacology and Toxicology, University of Southern California, Los Angeles, CA 90033, USA

**Keywords:** pancreatic cancer, cancer stem cells, drug resistance, self-renewal, Wnt signaling

## Abstract

Background: Although canonical Wnt signaling is known to promote tumorigenesis in pancreatic ductal adenocarcinoma (PDAC), a cancer driven principally by mutant *K-Ras*, the detailed molecular mechanisms by which the Wnt effector β-catenin regulates such tumorigenesis are largely unknown. We have previously demonstrated that β-catenin’s differential usage of the Kat3 transcriptional coactivator cyclic AMP-response element binding protein-binding protein (CBP) over its highly homologous coactivator p300 increases self-renewal and suppresses differentiation in other types of cancer. Aim/methods: To investigate Wnt-mediated carcinogenesis in PDAC, we have used the specific small molecule CBP/β-catenin antagonist, ICG-001, which our lab identified and has extensively characterized, to examine its effects in human pancreatic cancer cells and in both an orthotopic mouse model and a human patient-derived xenograft (PDX) model of PDAC. Results/conclusion: We report for the first time that *K-Ras* activation increases the CBP/β-catenin interaction in pancreatic cancer; and that ICG-001 specific antagonism of the CBP/β-catenin interaction sensitizes pancreatic cancer cells and tumors to gemcitabine treatment. These effects were associated with increases in the expression of let-7a microRNA; suppression of *K-Ras* and survivin; and the elimination of drug-resistant cancer stem/tumor-initiating cells.

## 1. Introduction

Pancreatic ductal adenocarcinoma (PDAC) is the most common neoplasm of the pancreas. Median survival for patients with PDAC is 5–8 months, with more than 95% of patients dying within 5 years or less. Indeed, PDAC is ranked the #4 cause of death from cancer among men and women in the West [1]. At present, surgical therapy affords the only chance for cure, yet such a “surgical cure” is limited to early stage pancreatic cancer. Currently, chemotherapy for advanced pancreatic cancer shows limited efficacy in regards to tumor control. Hence, there is a necessity for more effective treatments for pancreatic cancer [1].

The Wnt signaling cascade, as it does in essentially all organ systems, plays an important role in pancreatic development in both mice and humans. However, as in many systems, it is unclear whether Wnt signaling regulates self-renewal or differentiation, or both. PDACs do not normally harbor mutations in classical Wnt regulators such as the adenomatous polyposis coli gene, *APC*. Yet, activating mutations of β-catenin, a downstream signaling partner for Wnt and loss-of-function mutations of *APC* have been observed in other pancreatic tumors including pancreatoblastomas [2], acinar cell carcinomas [3] and solid pseudo papillary tumors [4]. More recently, in vitro and in vivo functional studies have demonstrated that activation of the canonical Wnt pathway affects tumorigenesis in PDAC, and a majority of PDAC is characterized as having an up-regulated Wnt/β-catenin transcriptional signature [5,6]. Furthermore, studies assessing protein and messenger RNA levels of PDX1 (a key transcriptional regulator in early-stage development of the pancreas and an embryonic progenitor cell marker) in human PDAC showed that PDX1 was most strongly expressed at the “leading edge” of a tumor where there is a high density of cancer stem/tumor-initiating cells (CSC/TIC) [7,8]. This population is highly refractory to therapy as explained by its relative quiescence, increased anti-apoptotic mechanisms, expression of multiple chemotherapeutic-resistant pumps, and dysregulation of developmental signaling pathways [9], including the Wnt signaling pathway [10].

Wnt signaling and in particular, the nuclear functions of β-catenin have been shown to be important in the maintenance, proliferation, and differentiation of stem cells, in embryonic and both normal adult somatic stem cells (SSC) as well as cancer stem cells (CSC) [11]. To generate a transcriptionally active complex, β-catenin recruits transcriptional Kat3 coactivators, cAMP response element binding protein (CREB)-binding protein (CBP) or its closely related homolog, p300 (E1A-binding protein, 300 kDa), as well as other components of the basal transcription machinery, leading to the expression of a host of downstream target genes [11]. Whereas CBP/β-catenin-mediated transcription is critical for stem cell/progenitor cell maintenance and proliferation, p300/β-catenin interaction mediates a transcriptional program that initiates differentiation and a decrease in cellular potency (the cell’s ability to differentiate into other cell types) [11].

Differentiation therapy for acute promyelocytic leukemia with all-*trans* retinoic acid was a breakthrough treatment for this leukemia [12]. However, attempts to extend this success have been only minimally successful on a broader scale in leukemia and even less so in solid tumors [13]. Our previous studies have demonstrated that a change from CBP/catenin-driven to p300/catenin-driven transcription is associated with the initiation of differentiation of both normal SSC and CSC [11]. Our studies have also demonstrated that this initiation of differentiation can be regulated by multiple catenins, including both β and γ [14]. We have previously demonstrated that ICG-001 can safely eliminate drug-resistant CSC/TIC via forced symmetric differentiation, thereby enhancing sensitivity to chemotherapy [11,15,16,17].

We chose to explore ICG-001 as potential differentiation therapy for PDAC based on the aforementioned and given that: (1) synchronous activation of Wnt and Ras (the latter of which is found to have activating mutations in ~95% of PDAC) signaling cascades has been observed in a variety of transgenic mouse models of cancers, such as those of colon [18], intestine [19], and liver [20]; (2) convergence of Wnt and *K-Ras* signaling pathways up-regulate the expression of a number of Wnt target genes [21,22,23] that promote tumorigenesis; (3) Wnt/β-catenin-driven maintenance of pancreatic CSC may be tied to resistance to chemotherapy [24].

Considering this background, our central hypothesis is that increased CBP/β-catenin-driven transcription, at the expense of p300/β-catenin-driven transcription, is associated with pancreatic cancer development, disease progression and chemotherapy resistance and maintenance of the CSC/TIC population. Furthermore, we propose and demonstrate that activation of mutant *K-Ras* and downstream MAPK kinase cascade biases differential coactivator usage towards increased CBP/β-catenin-mediated transcription. Coactivator imbalance in the Wnt/β-catenin signaling cascade results in the expansion of pancreatic cancer CSC/TIC, which are associated with drug resistance, metastases and poor prognosis in patients with pancreatic cancer [24]. We herein report for the first time that *K-Ras* activation increases CBP/β-catenin interaction in pancreatic cancer and that differentiation therapy specifically targeting CBP/β-catenin interaction with ICG-001 can safely eliminate CSC/TIC in a patient-derived xenograft (PDX) model, thereby sensitizing the tumor to gemcitabine treatment. These effects were associated with increases in expression of let-7a microRNA; suppression of *K-Ras* and survivin; and elimination of drug-resistant CSC/TICs.

## 2. Results

### 2.1. K-Ras Mutations Increase Wnt/β-Catenin Signaling in Pancreatic Cancer Cells

**Figure 1:**
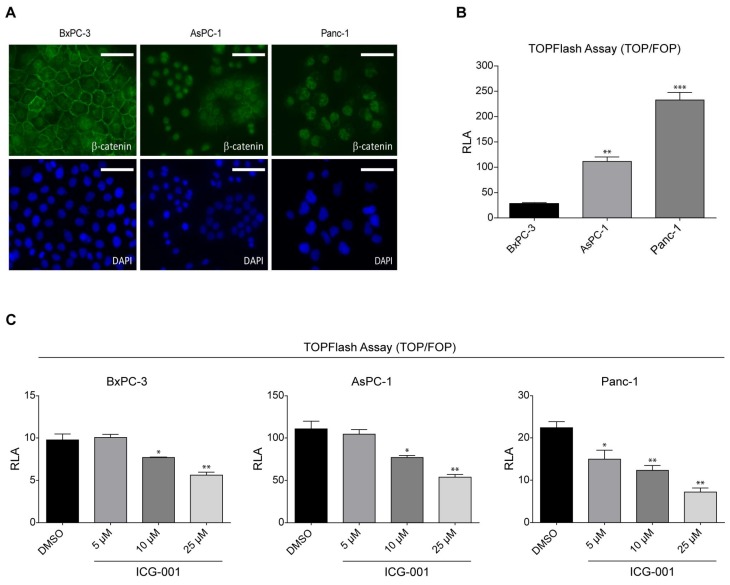
*K-Ras* activating mutation increases Wnt/β-catenin signaling in human pancreatic cancer cells. (**A**) BxPC-3, AsPC-1, and PANC-1 pancreatic cancer cells were assayed by immunofluorescence to determine cellular localization of β-catenin. BxPC-3 cells, expressing wild type *K-Ras*, showed distinct membrane localization of β-catenin and weak, diffuse nuclear and cytoplasmic staining (DAPI, counterstain). However, in *K-Ras* mutant cells AsPC-1 and PANC-1 there was virtually no membrane-associated β-catenin. AsPC-1 cells showed diffuse cytoplasmic staining and strong nuclear staining for β-catenin, whereas PANC-1 cells demonstrated strong nuclear staining with limited cytoplasmic staining. Scale bar = 100 µm. (**B**) TOPFlash reporter gene assay was used to evaluate Wnt/β-catenin transcriptional activation in BxPC-3, AsPC-1, and PANC-1 cells. BxPC-3 cells exhibited weak Wnt/TCF/β-catenin-driven luciferase expression. However, AsPC-1 and PANC-1 cells demonstrated enhanced TOPFlash activity. n = 3, ** *p* < 0.01, *** *p* < 0.001, compared to BxPC-3. RLA, relative luciferase activity. (**C**) Treatment with ICG-001, a specific, small molecule inhibitor of CREB-binding protein (CBP)/β-catenin interaction, demonstrated dose-dependent decrease in TOPFlash activity in BxPC-3, AsPC-1, and PANC-1 cells. n = 3, * *p* < 0.05, ** *p* < 0.01, compared to DMSO control. RLA, relative luciferase activity.

We initiated our investigations using 3 human pancreatic cancer cell lines BxPC-3 (*K-Ras* wild type), AsPC-1 (*K-Ras* mutant), and PANC-1 (*K-Ras* mutant). We initially focused our attention on the localization of β-catenin in these cell lines. BxPC-3 cells, which express wild type *K-Ras*, showed distinct membrane localization of β-catenin as judged by immunofluorescence (IF) and relatively weak and diffuse nuclear and cytoplasmic staining (with DAPI counterstain) (Figure 1A). In contrast, in both the *K-Ras* mutant cell lines AsPC-1 and PANC-1 there was virtually no evidence of membrane associated β-catenin. AsPC-1 cells showed diffuse cytoplasmic staining and relatively strong nuclear staining for β-catenin, whereas PANC-1 cells demonstrated strong nuclear staining with very limited cytoplasmic staining (Figure 1A).

β-catenin’s localization to the nucleus is a prerequisite for activation of canonical Wnt signaling. To evaluate Wnt/β-catenin transcriptional activation in the 3 cell lines, we used the TOPFlash reporter gene assay. TOPFlash is a luciferase reporter readout for β-catenin/TCF-mediated transcriptional activation. BxPC-3 cells exhibited relatively weak Wnt/TCF/β-catenin-driven luciferase expression (Figure 1B). As anticipated, both of the *K-Ras* mutant cell lines that exhibited significantly greater nuclear β-catenin localization, also demonstrated significantly enhanced TOPFlash activity (AsPC-1 ~4X and PANC-1 ~10X), respectively (Figure 1B).

ICG-001 is a specific small molecule antagonist of the CBP/β-catenin interaction, which binds specifically to the amino terminus of Kat3 coactivator CBP without binding to the highly homologous coactivator p300 [25,26]. ICG-001 disrupts expression of a subset of Wnt/β-catenin signaling and decreases TOPFlash activation in colorectal cancer cells that display constitutively high activation of canonical Wnt/β-catenin signaling [25]. As anticipated, treatment with ICG-001 demonstrated a dose-dependent decrease in TOPFlash/luciferase activity in all three cell lines (Figure 1C).

### 2.2. ICG-001 Effects a Change in β-Catenin Coactivator Usage in Pancreatic Cancer Cells

**Figure 2:**
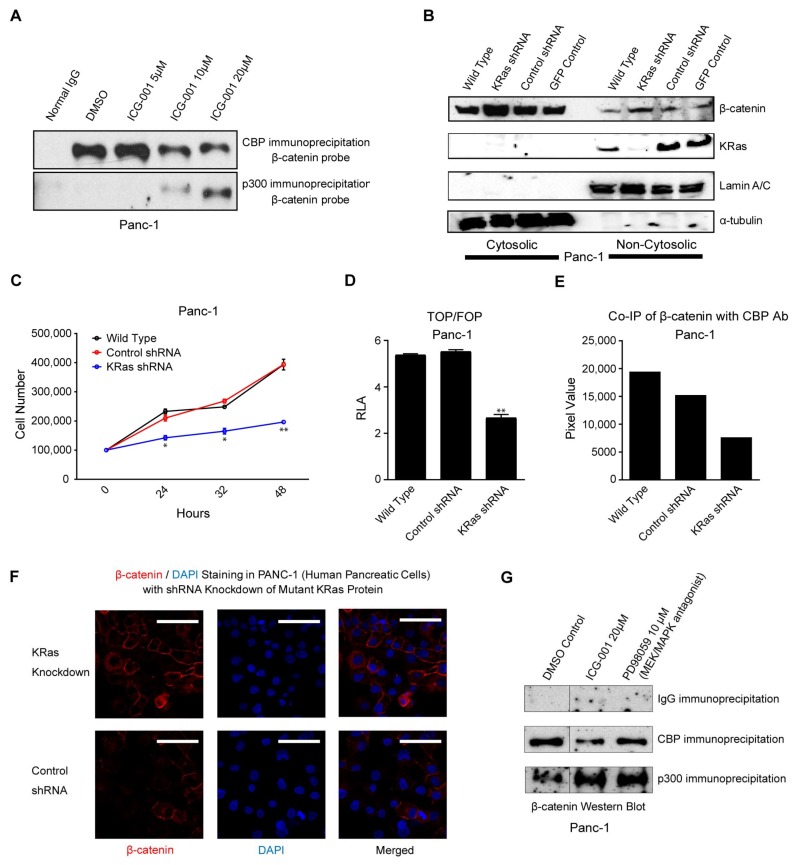
ICG-001 antagonizes CBP/β-catenin interaction in human pancreatic cancer cells, whereas *K-Ras* activation increases CBP/β-catenin interaction. (**A**) Immunoprecipitation of nuclear lysates of PANC-1 cells with CBP specific antibody or p300 specific antibody with subsequent immunoblotting for β-catenin was performed. In the absence of ICG-001, essentially all β-catenin is associated with CBP. Treatment with ICG-001 decreased CBP associated β-catenin with a concomitant increase in β-catenin immunoprecipitated with p300. (**B–F**) *K-Ras* specific lentiviral shRNA was used to deplete *K-Ras* in PANC-1 cells. Suppression of *K-Ras* increased cytoplasmic β-catenin without affecting non-cytoplasmic β-catenin as shown by immunoblot (**B**), decreased cell proliferation, n = 2, * *p* < 0.05, ** *p* < 0.01, compared to control (**C**), decreased TOPFlash activity, n = 4 technical replicates, ** *p* < 0.01, compared to control, RLA, relative luciferase activity (**D**), decreased the amount of β-catenin associated with CBP as shown by co-immunoprecipitation (**E**), and increased membrane-associated β-catenin as shown by immunofluorescence, Scale bar = 100 µm (**F**). (**G**) Similar to effects of ICG-001, pharmacologic treatment with MEK/MAPK antagonist PD98059 decreased CBP/β-catenin interaction and concomitantly increased p300/β-catenin interaction, as assessed by immunoblot. Dividing line indicates splice junction.

ICG-001 specifically blocks binding of β-catenin to coactivator CBP and thereby forces β-catenin to partner with the highly homologous coactivator p300 for gene transcription [25,26]. Differential coactivator usage has been demonstrated to be causal for the initiation of differentiation and a loss in cellular potency in both stem cells and CSC/TIC [15,16,17,26,27]. To confirm that the effects of ICG-001 in pancreatic cancer cells were associated with the same biochemical mechanism of action, we performed a β-catenin co-immunoprecipitation experiment. PANC-1 cells were treated with either DMSO control or increasing concentrations of ICG-001. Immunoprecipitation of freshly prepared nuclear lysates either with CBP specific antibody or p300 specific antibody with subsequent immunoblotting for β-catenin was performed. As can be seen (Figure 2A), in the absence of ICG-001 (DMSO lane 2), essentially all of β-catenin is associated with CBP. However, no changes in total nuclear β-catenin, CBP, and p300 levels were obvious with ICG-001 treatment (Figure S1). This situation is similar to that previously observed in colorectal cancer cell lines [25] as well as in primary colon cancer patient samples [28] and also in embryonic stem cells, both mouse [29] and human [27]. Treatment with increasing concentrations of ICG-001 decreased CBP-associated β-catenin with a concomitant increase in β-catenin immunoprecipitated with p300 (Figure 2A lanes 3–5).

### 2.3. K-Ras Activation Increases the CBP/β-Catenin Interaction

We next decided to investigate potential mechanisms whereby activated *K-Ras* affects the Wnt signaling pathway, particularly in the choice of Kat3 coactivator usage by β-catenin. We first utilized specific shRNA to knockdown *K-Ras* in PANC-1 cells. PANC-1 cells expressed abundant *K-Ras*, which was substantially depleted by *K-Ras* specific shRNA but was not diminished by control shRNAs (Figure 2B). Compared to control PANC-1 cells, the knockdown of *K-Ras* by shRNA substantially increased cytoplasmic β-catenin without substantially affecting non-cytoplasmic β-catenin (Figure 2B), and dramatically decreased cell proliferation (Figure 2C), decreased TOPFlash activity by approximately 50% (Figure 2D), and also decreased the amount of β-catenin associated with CBP compared to non-treated cells or control shRNA-treated cells (Figure 2E). Indeed, we hypothesize that reduced proliferation, as manifested by a smaller number of *K-Ras* shRNA cells versus wildtype PANC-1 cells (Figure 2C), is due to decreased CBP/β-catenin binding (Figure 2E) and the associated decreased β-catenin/TCF-mediated activation (Figure 2D) of cellular proliferation pathways, despite no apparent reduction in nuclear β-catenin levels. Knockdown of *K-Ras* also increased membrane-associated β-catenin (Figure 2F).

Mutant *K-Ras* induces downstream activation of ERK/MAP kinase-signaling cascade. To test whether *K-Ras* downstream activation of the MAPK cascade could play a role in modulating β-catenin coactivator usage, we investigated differential β-catenin association with coactivators CBP and p300 in PANC-1 cells treated with selective MEK inhibitor PD90859. Treatment with PD90859 reduced proliferation of PANC-1 cells (data not shown), although no changes in total nuclear β-catenin, CBP, and p300 levels were obvious with PD90859 or ICG-001 treatment (Figure S2). Co-immunoprecipitation was subsequently performed with specific antibody against CBP with IgG used as the negative control. As shown (Figure 2G), similar to the effects of ICG-001, pharmacologic inhibition of MEK decreased CBP/β-catenin interaction and simultaneously increased p300/β-catenin interaction. We conclude that oncogenic *K-Ras* can affect Wnt/β-catenin signaling, as evidenced by decreased TOPFlash activity in *K-Ras* shRNA cells (Figure 2D), decreased CBP/β-catenin interaction in *K-Ras* shRNA cells (Figure 2E) and in PD98059-treated cells (Figure 2G), increased p300/β-catenin binding in PD98059-treated cells (Figure 2G), and increased membrane-associated β-catenin in *K-Ras* shRNA cells (Figure 2F).

### 2.4. Decreasing CBP/β-Catenin Interaction While Increasing p300/β-Catenin Interaction Results in Reduced Expression of Genes Associated with Poor Prognosis

**Figure 3:**
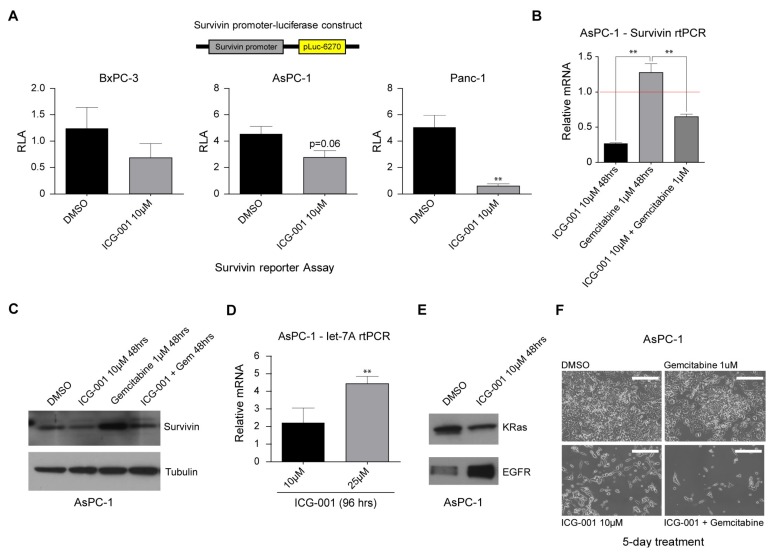
ICG-001 counteracts survivin and *K-Ras* pathways, sensitizing human pancreatic cancer cells to conventional therapy. (**A**) The survivin/luciferase promoter construct (pLuc-6270) was used to evaluate the effects of ICG-001 in BxPC-3, AsPC-1, and PANC-1 cells. Treatment with ICG-001 decreased survivin/luciferase activity in all three cell lines. n = 3, ** *p* < 0.01, compared to DMSO control. RLA, relative luciferase activity. (**B**,**C**) ICG-001 decreased survivin message (relative mRNA expression for DMSO control indicated by red horizontal line) (**B**) and protein levels (**C**) in AsPC-1 cells, as shown by RT-qPCR and immunoblot, respectively. However, treatment with gemcitabine caused an increase in survivin message and protein levels. Importantly, ICG-001 counteracted the gemcitabine-mediated increase in survivin message and protein levels. n = 3, ** *p* < 0.01. D. ICG-001 treatment of AsPC-1 cells for 96 h increased expression of let-7a as assessed by RT-qPCR. n = 3, ** *p* < 0.01, compared to DMSO control. (**E**) ICG-001 treatment of AsPC-1 cells for 48 h decreased K-Ras protein levels but increased EGFR levels as assessed by immunoblot. (**F**) Compared to treatment with a single agent, a combination of gemcitabine and ICG-001 treatment of AsPC-1 cells for 5 days dramatically decreased cell growth and viability as assessed by morphology. Scale bar = 500 µm.

Many important genes involved in oncogenesis, drug resistance and metastasis, for example, survivin/BIRC5, the number 4 transcriptome up-regulated in cancer [30], are regulated via activation of Wnt/β-catenin-mediated transcription [26]. We have previously shown that such genes are highly dependent on the use of coactivator CBP and that antagonizing CBP/β-catenin interaction blocks recruitment of CBP to their respective promoters, thereby significantly decreasing the expression of these genes [15,17,25,31,32]. Overexpression of survivin in a variety of human cancers, including PDAC, is known to mediate the proliferation of neoplastic cells, growth of cancers, and resistance of cancers to chemotherapy. Therefore, inhibiting survivin expression should be beneficial for treating pancreatic cancer. We initially utilized the survivin/luciferase promoter construct (pLuc-6270) [31] to evaluate the effects of ICG-001 in the pancreatic cancer cell lines. All of the lines demonstrated significant survivin/luciferase activity after transient transfection of the survivin reporter construct. Treatment with 10 µM ICG-001 significantly decreased survivin/luciferase activity in both BxPC-3 and AsPC-1 cells and dramatically so in PANC-1 cells (Figure 3A). We next examined expression of survivin message and protein in the pancreatic cancer cell line AsPC-1 in the presence or absence of the deoxycytidine analog gemcitabine, which since 1996 has been approved by the FDA as a first-line treatment for patients with PDAC, and/or ICG-001. We were particularly interested in testing combination treatment with ICG-001 and gemcitabine, given that gemcitabine is known to induce survivin expression in pancreatic cancer cells [33]. As anticipated, 10 µM ICG-001 significantly decreased survivin message (Figure 3B) and protein levels (Figure 3C) in AsPC-1 cells. In contrast, treatment of AsPC-1 cells with 1 µM gemcitabine caused an increase in survivin message and protein levels (Figure 3B,C). Importantly, ICG-001 could counteract gemcitabine-mediated increases in both survivin message and protein levels (Figure 3B,C). Similar results were obtained in PANC-1 cells (data not shown). We next utilized the WaferGen SmartChip system for high-throughput, real-time polymerase chain reaction (PCR) studies of AsPC-1 cells grown in the presence or absence of ICG-001 for 96 h. Of the approximately 1000 genes represented on the chip, 40 were down-regulated by 50% or more after ICG-001 treatment, including *CCNB1* and *ALDH1A* (Table S1), genes associated with cell cycle regulation and pancreatic cancer stem/progenitor cells [34], respectively. As anticipated, ICG-001 treatment down-regulated survivin/BIRC5 expression ~2 fold on the array as had been observed by individual reverse transcription quantitative polymerase chain reaction (RT-qPCR) analysis. The expression of 80 target genes on the chip was increased by greater than two-fold. Interestingly, 3 members of the epidermal growth factor receptor pathway Erbb3, Erbb2 and EGFR were up-regulated (2.3, 4.5 and 3.8-fold respectively) after ICG-001 treatment. We have previously observed up-regulation of a variety of growth factor pathways after antagonism of CBP/catenin interaction in vitro (e.g., BCR-ABL, EGFR, Met) [17] (and Michael Kahn, unpublished observations). This type of “oncogenic addiction” makes tumor cells particularly vulnerable to pathway specific targeted chemotherapy after CBP/catenin antagonist treatment [15,16,17].

### 2.5. ICG-001 Induces let-7 miRNA Expression and Suppresses K-Ras Protein Levels while Increasing EGFR Protein Levels in Pancreatic Cancer Cells

We have previously demonstrated that a switch in coactivator usage by β-catenin from CBP to p300 initiates a change in gene expression associated with initiation of differentiation in stem/progenitor cell populations and epigenetic changes [15,16,17,29,31,35]. MicroRNAs (miRNAs) are known to play important roles in controlling protein translation and are implicated in regulating cellular fate and the behavior of progenitor/stem cells. We therefore decided to investigate the effects of ICG-001 on miRNA expression of members of the let-7 family as Li et al. had reported that the expression of a number of let-7 family members was significantly down-regulated in gemcitabine-resistant pancreatic cancer cells [36]. Treatment of AsPC-1 and PANC-1 cells with ICG-001 for 24 h to 96 h led to increased expression of let-7a over time (approximately 1.5-fold to 4.5-fold) (Figure 3D and data not shown). The let-7 family of miRNAs has been shown to play an important role in the differentiation of stem cells [37] and particularly in regulation of the *RAS* oncogene, and lower levels of let-7 are associated with a poor outcome [36]. Interestingly, in association with the increase in let-7a miR expression, although there is no change in K-Ras message levels, there was a significant decrease in K-Ras protein levels in treated AsPC-1 cells and concomitantly a significant increase in EGFR protein levels (Figure 3E). We conclude that treatment with ICG-001 increases expression of let-7a microRNA consistent with an increase in differentiation and a decrease in K-Ras protein levels, which overall should enhance sensitivity to chemotherapy.

### 2.6. ICG-001 Effectively Sensitizes Pancreatic Cancer Cells to Conventional Chemotherapy

Five days of treatment with gemcitabine alone demonstrated a minimal effect on cell growth and morphology of AsPC-1 cells. Interestingly, ICG-001 (10 µM) had a more significant effect on cell growth and also affected cell morphology. In combination, gemcitabine and ICG-001 dramatically decreased cell growth and viability (Figure 3F). We conclude that there was increased efficacy in vitro, when utilizing a combination of the CBP/catenin antagonist ICG-001 with cytotoxic (gemcitabine) chemotherapy than any single agent.

### 2.7. Efficacy of CBP/Catenin Antagonists in In Vivo Models

**Figure 4:**
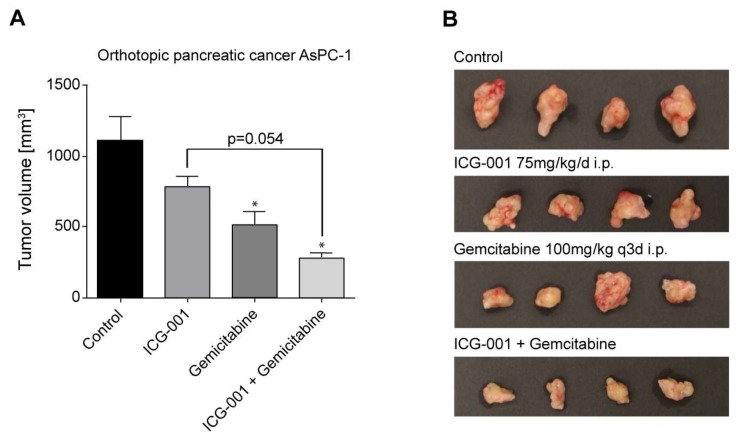
Combination ICG-001 and gemcitabine treatment suppresses pancreatic tumor growth in an orthotopic mouse model of PDAC. 1 × 10^5^ AsPC-1 cells were injected into the pancreas of nu/nu mice. After 5 days, treatment was initiated with 4 groups, i.e., saline control, ICG-001 75 mg/kg/day i.p., gemcitabine 100 mg/kg/day twice a week i.p., or the combination. Treatment was continued for 6 weeks at which point mice were sacrificed and tumors examined. (**A**) Tumor volume was decreased by ICG-001 alone, but not significantly. Gemcitabine alone effected approximately 50% reduction in tumor volume compared to control. Combination of ICG-001 and gemcitabine caused further decrease in tumor growth with nearly 75% reduction in volume. n = 4, * *p* < 0.05, compared to control. (**B**) Volume of tumors was clearly affected as judged visually, particularly in the combination treatment group.

Having determined that ICG-001 alone slowed the growth of pancreatic cancer cells and was effective in combination with gemcitabine in vitro, we next sought to test the effects of ICG-001 either alone or in combination with gemcitabine in vivo in mouse models of pancreatic cancer. We first evaluated ICG-001, gemcitabine or the combination in an orthotopic pancreatic cancer model using AsPC-1 cells [38]. 1 × 10^5^ AsPC-1 cells were injected into the pancreas of nu/nu mice. After 5 days, treatment was initiated. The study involved 4 groups (saline control, ICG-001 75 mg/kg/day i.p., gemcitabine 100 mg/kg/day twice a week i.p., or the combination). Treatment was continued for 6 weeks at which point mice were euthanized and tumors examined. Tumor volume was somewhat decreased by ICG-001 alone, but not significantly. Gemcitabine alone effected an approximately 50% reduction in tumor volume compared to control (Figure 4A). The combination of ICG-001 with gemcitabine caused a further decrease in tumor growth with a nearly 75% reduction in volume (Figure 4A). The volume of the tumors was also clearly affected as judged visually, particularly in the combination group (Figure 4B).

**Figure 5:**
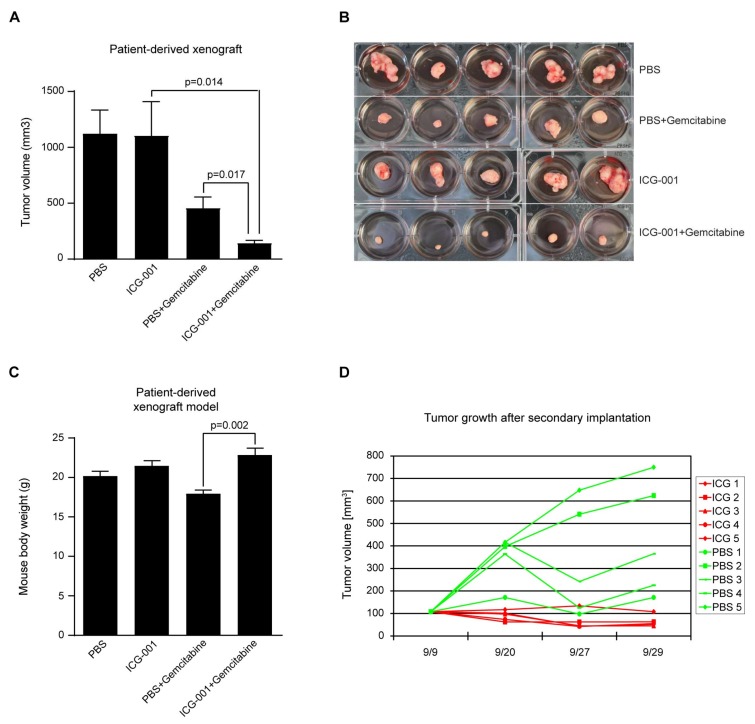
Combination ICG-001 and gemcitabine treatment suppresses human patient-derived PDAC in a mouse xenograft model; ICG-001 effectively targets tumor-initiating cells. (**A**–**C**) Patient-derived PDAC was implanted subcutaneously into the flanks of immunocompromised NOD/SCID/IL2rγ−/− mice. Two weeks after primary implantation, treatment was initiated with 4 groups, i.e., saline control, ICG-001 50 mg/kg/day s.c., gemcitabine 100 mg/kg/day twice a week i.p., or the combination. Treatment was continued for 3 weeks at which point mice were sacrificed and tumors examined. Whereas ICG-001 alone failed to slow growth of tumors, gemcitabine alone significantly slowed tumor growth. However, combination of ICG-001 plus gemcitabine most dramatically decreased tumor growth as evidenced by tumor volume and visual inspection (**A**,**B**). ICG-001 ameliorated gemcitabine toxicity as judged by increased body mass among mice in the combination treatment group versus the gemcitabine-only treatment group (**C**). n = 5. (**D**) After secondary implantation of human PDAC was performed without any further treatment, tumors previously subjected to ICG-001 treatment (during primary implantation) failed to grow, whereas tumors previously subjected to phosphate buffered saline (PBS) treatment grew similarly to the first implantation, indicating that ICG-001 targets tumor-initiating cells. n = 5.

To further evaluate the efficacy of these agents in pancreatic cancer, we utilized a primary patient derived xenograft model generated from a resected PDAC, and implanted tumors subcutaneously into the flanks of highly immunocompromised NOD/SCID/IL2rγ−/− mice. We again used 4 groups (saline control, ICG-001 50 mg/kg/day s.c., gemcitabine 100 mg/kg/day twice a week i.p., or the combination). Similar to the orthotopic model, ICG-001 alone had a very limited effect on the growth of the tumors. Gemcitabine significantly slowed the growth of the tumor xenografts. However, the combination of ICG-001 plus gemcitabine dramatically decreased the volume of the tumors (Figure 5A,B). Gemcitabine, just as in humans, has dose-limiting toxicities in mice. The toxicity of gemcitabine in our model manifested itself as reduced weight in mice treated with gemcitabine alone versus either phosphate buffered saline (PBS) or ICG-001 treated mice. Importantly, rather than increasing toxicity in combination with gemcitabine, ICG-001 ameliorated gemcitabine toxicity as judged by higher body weight in the combination group versus the gemcitabine-only treated mice (Figure 5C). Interestingly, although ICG-001 alone did not inhibit the growth of the xenograft upon initial transplantation, when a secondary implantation was performed without any further treatment, the ICG-001 treated tumor failed to grow, whereas the PBS-treated tumor grew similarly to the first passage (Figure 5D). These results are interpreted as demonstrating the ability of ICG-001 to safely and selectively target the PDAC CSC/TIC population. As a single agent, this is manifested as blocking tumor outgrowth after secondary transplantation, whereas it dramatically sensitizes the primary tumor to gemcitabine, while concomitantly ameliorating gemcitabine toxicity.

## 3. Discussion

Pancreatic cancer is amongst the most highly aggressive and lethal cancers. It is characterized by rapid proliferation, metastasis, and resistance to chemotherapy. As established by the randomized Phase III trial of Burris et al. [39], gemcitabine monotherapy had been the standard of care for treating metastatic disease with a median survival of 5.65 months. Combination therapy using gemcitabine with oxaliplatin, cisplatin, or irinotecan have not been shown to effect significant survival benefit in comparison with gemcitabine monotherapy [40]. A trial of gemcitabine combined with oral small molecule EGFR tyrosine kinase inhibitor erlotinib demonstrated a modest increase in survival for the combination, with median survival increasing from 5.9 to 6.2 months [41]. Most recently, a combination of Abraxane plus gemcitabine was approved by the FDA based upon improved overall survival from 6.7 to 8.5 months in a randomized international trial enrolling 861 patients with metastatic pancreatic cancer [42]. However, there is still a significant need to develop more efficacious treatment strategies for PDAC.

More than 90% of PDACs involve mutations in the oncogene *K-Ras* [43]. *K-Ras* plays an important role in pancreatic cancer as demonstrated via RNA interference/knockdown (RNAi) studies [44]. MAPK inhibition downstream of *K-Ras* activation suppresses proliferation of PDAC cell lines [44]. Furthermore, subjects with *K-Ras*-negative tumors demonstrate improved survival after radiation therapy than those with tumors carrying the mutated, activated oncogene [45]. Mutations in classic Wnt regulators, such as APC or β-catenin, which are frequently found in other GI cancers, are uncommon in PDAC. However, more recent in vitro and in vivo functional studies show that the canonical Wnt signaling pathway effects PDAC tumorigenesis, and the majority of PDAC is characterized as having an up-regulated Wnt/β-catenin transcriptional signature [5,6]. Synchronous activation of Wnt and Ras signaling cascades in a variety of transgenic mouse models of cancers, including colon [18], intestine [19], and liver [20], converge to up-regulate numerous genes (e.g., cyclooxygenase-2, c-myc and interleukin-8) [21,22,23], which promote tumorigenesis.

**Figure 6:**
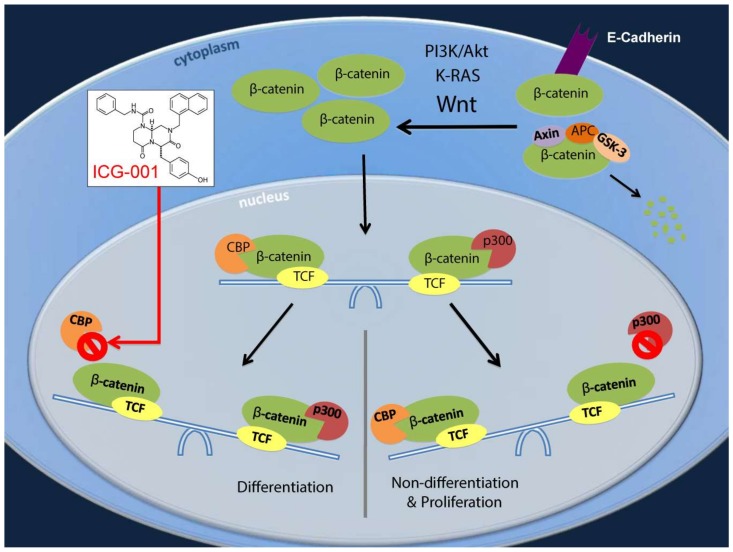
ICG-001 specifically blocks the CBP/β-catenin interaction. As a result, there is a shift from CBP/β-catenin pathways, which effect the proliferation/potency (non-differentiation) transcriptional program, to p300/β-catenin pathways, which effect the differentiation transcriptional program (with loss of stemness). ICG-001 modulates crosstalk between Wnt/β-catenin and *K-Ras* signaling pathways which converge at the level of CBP/β-catenin versus p300/β-catenin interaction. Thus, ICG-001 can sensitize pancreatic cancer to conventional therapy, via suppression of survivin, induction of let-7a, and eradication of highly drug-resistant tumor-initiating cells.

We now show that PDAC cell lines AsPC-1 and PANC-1, which carry an activating *K-Ras* mutation, evidence an enhanced activated Wnt/β-catenin transcriptional signature, as judged by the presence of dramatically increased cytoplasmic and nuclear β-catenin, increased TOPFlash activation and expression of the Wnt/β-catenin target gene survivin/BIRC5, compared to BxPC-3 cells which carry wild type *K-Ras*. Furthermore, we demonstrate that the nuclear β-catenin in these cells is predominantly associated with Kat3 coactivator CBP and not the highly homologous coactivator p300, and just as we have previously demonstrated in colorectal cancer cells [25] and primary patient derived colon cancer [28] and leukemia-initiating cells in both ALL [15] and CML [17], ES both mouse [29] and human [27], cells undergoing an EMT-like process [32] and normal adult stem/progenitor cells undergoing differentiation [46], ICG-001 by specifically binding to the N-terminus of CBP, antagonizes CBP/β-catenin interaction, thereby inducing a switch in β-catenin coactivator usage to p300 (Figure 2A). This is fully consistent with our model concerning the role of differential Kat3 coactivator usage in self-renewal and differentiation in stem and progenitor cells (Figure 6) [11]. Similarly, pharmacologically antagonizing MAPK cascade downstream of activated *K-Ras*, or knockdown of *K-Ras* in these cells, also decreases Wnt/CBP/β-catenin interaction. However, details of how mechanistically KRAS/MAPK activation enhances CBP/catenin interaction are not known. The amino terminal regions of both CBP and p300 contain a large number of serine and threonine residues that are potential phosphorylation sites [29,46]. We have only begun to investigate the rapid and reversible kinase/phosphatases cascades that modulate Kat3 coactivator protein/protein interactions. We previously demonstrated that p300 serine 89 (p300S89) phosphorylation was important for differentiation of both embryonic [29] and adult stem cells [46] and that the small molecule PP2A antagonist IQ-1, may indirectly block phosphorylation of p300S89 by blocking the dephosphorylation of p300S90 [29,46]. Interestingly, p300S90 is adjacent to P91, making it a consensus sequence phosphorylation site for both MAPK and CDK family kinases. We speculate that phosphorylation of p300S90 via the MAPK cascade could play a role in enhancing CBP/catenin interaction and will be addressed in future investigations.

We also demonstrate that ICG-001 decreased TOPFlash expression in treated cells in a dose-dependent manner. We have previously demonstrated that survivin, the number 4 transcript up-regulated in cancer [30], is a Wnt/CBP/β-catenin target in colorectal cancer cells and colorectal cancer patient circulating tumor cells [25,31] and its expression is decreased upon treatment with specific CBP/β-catenin antagonists. Survivin is a member of the Inhibitor of Apoptosis (IAP) gene family which prevents cell death and regulates progression of mitosis [47]. Survivin is a 16 kDa protein which is expressed during development of the embryo, but is essentially not detectable in terminally differentiated tissues of the adult and not necessary for normal differentiated cell viability [47]. Survivin is known to be overexpressed in many cancers and increased expression of survivin has been correlated with poor prognosis. Similar to results obtained in colorectal, breast and prostate cancer [25,31], PDAC cell lines AsPC-1 and PANC-1 with mutant K-Ras demonstrate high levels of expression of survivin. Treatment with ICG-001 decreased expression of survivin in both of these PDAC cell lines. Increased survivin expression is also associated with resistance to chemotherapeutic agents [47]. We demonstrate that, whereas treatment with ICG-001 decreases survivin expression at both the message and protein levels, treatment with gemcitabine increases both the survivin message and protein. Importantly, treatment with gemcitabine in combination with ICG-001 decreased survivin expression significantly compared with gemcitabine treatment alone. We speculate that this reduction in survivin is at least partially associated with the sensitization of the PDAC cells to gemcitabine in the presence of ICG-001. In addition, we found that ICG-001 treatment induced EGFR expression and thus may sensitize PDAC to targeted therapy with EGFR tyrosine kinase inhibitor erlotinib. We further demonstrated that ICG-001 increases expression of microRNA let-7a, consistent with an increase in differentiation and with a concomitant decrease in K-Ras protein levels.

Finally, we have demonstrated that combination therapy with ICG-001 and gemcitabine has significant beneficial effects in both an orthotopic PDAC mouse model and a PDAC PDX model. An earlier report by Arensman et al. stated that although ICG-001 (5 mg/kg/day 6 days per week) demonstrated significantly increased survival over the vehicle, the combination of ICG-001 and gemcitabine increased survival but not significantly over gemcitabine alone [48]. However, combining an increased dose of ICG-001 (i.e., 50–75 mg/kg versus 5 mg/kg), which we have previously shown to be safe and efficacious in multiple preclinical models [15], with gemcitabine (100 mg/kg versus 25 mg/kg) demonstrated that combined treatment effected a dramatic reduction in tumor growth in both our orthotopic PDAC and PDX models compared to either single agent (Figure 4A,B and Figure 5A,B). Interestingly, the CBP/catenin antagonist ICG-001 also abrogated body weight loss due to gemcitabine toxicity when given in combination in the PDX model (Figure 5C) [11]. The ability to target a subset of cells within a heterogeneous pancreatic tumor that display an activated Wnt/β-catenin signaling phenotype, which at least partially overlaps with the CSC/TIC population, utilizing specific small molecule antagonists of CBP/β-catenin interaction, especially in combination with existing chemotherapy agents (e.g., gemcitabine), offers a potentially attractive approach to safely treat highly aggressive and resistant pancreatic tumors.

Long-lived, essentially immortal, somatic stem cells (SSC) have an intrinsic preference to divide asymmetrically, whereas cancer stem cells (CSC) preferentially divide symmetrically [49,50]. Specific CBP/catenin antagonists take advantage of this intrinsic difference between SSC and CSC. The extremely high biochemical selectivity of ICG-001 for the N-terminus of its molecular target CBP and the unique evolutionarily defined properties of the two Kat3 coactivators CBP and p300, allow CBP/catenin antagonists to safely force CSC symmetric differentiative divisions, thereby stochastically eliminating quiescent CSC, whereas asymmetric divisions per force always leave one SSC intact in its niche [11].

The second-generation specific CBP/catenin antagonist PRI-724 completed a Phase Ia study in 2013 and demonstrated a very favorable toxicity profile. PRI-724 also demonstrated the ability to reduce survivin expression in circulating tumor cells in a dose-dependent manner [51]. More recently, in a Phase Ib study of patients with advanced PDAC, PRI-724 plus gemcitabine, as second-line therapy after fluorouracil or folinic acid/fluorouracil/oxaliplatin treatment failure, demonstrated stabilization of disease in 40% of patients, with 62.5% of patients who had elevated CA19-9 levels (at baseline) showing a decrease in this marker of >30% [52]. Further clinical investigations should inform us regarding optimization of dosage regimen and the clinical efficacy of combining CBP/catenin antagonists with either cytotoxic or targeted chemotherapeutics to treat PDAC.

## 4. Materials and Methods

### 4.1. Cancer Cell Lines

Human pancreatic cancer cell lines BxPC-3, AsPC-1, and PANC-1 were obtained from American Type Culture Collection (Manassas, VA, USA).

### 4.2. Pharmacologic Agents

Specific, small molecule CBP/β-catenin antagonist ICG-001 identified by our research group as previously described [25] was used at concentrations as indicated. MEK/MAPK antagonist PD98059 (Enzo Life Sciences, Farmingdale, NY, USA, catalog #: BML-EI360-0005) and chemotherapeutic gemcitabine (Enzo Life Sciences, catalog #: ALX-480-101) were used at concentrations as indicated.

### 4.3. Orthotopic Pancreatic Cancer in Nude Mice

These experiments were performed with IACUC (Protocol #11471) approval. AsPC-1 pancreatic cancer cells were stably transfected with a lentiviral luciferase construct. The AsPC-1-luciferase cells (1 × 10^5^/50 μL PBS) were orthotopically implanted into the pancreata of 10-week-old female nu/nu mice (Jackson Laboratory, Bar Harbor, ME, USA). Tumor engraftment was confirmed by bioluminescence imaging (IVIS200 Xenogen-Imaging system; Caliper Life Science, Hopkinton, MA, USA) 15 min after luciferin injection (1.5 mg/mouse i.p.). Mice were randomized to the following treatment groups: control, ICG-001 (75 mg/kg/day i.p.), gemcitabine (100 mg/kg i.p. twice a week for six weeks), combination of ICG-001 and gemcitabine. Treatment with ICG-001 was initiated on day 5 after tumor cell implantation. Chemotherapy with gemcitabine was initiated two days later, i.e., at day seven after tumor cell implantation. Mice were evaluated daily for signs of side effects due to therapy and tumor growth. Body weight was measured twice weekly. All mice were euthanized and underwent necropsy after six weeks of treatment. Therapeutic effects were judged according to tumor volume.

### 4.4. Human Patient-Derived Xenograft Model of Pancreatic Ductal Adenocarcinoma (PDAC)

These experiments were performed with IRB (Protocol #HS-06-00678) and IACUC (Protocol #11471) approval. Patient-derived PDAC tissue (~100 mm^3^, i.e., roughly 5 mm per dimension) was implanted subcutaneously into the flanks of 10-week-old female immunocompromised NOD/SCID/IL2rγ−/− (NSG) mice (Jackson Laboratory). Two weeks after primary implantation, treatment was initiated with 4 groups (n = 5 mice per group), i.e., saline control, ICG-001 50 mg/kg/day s.c., gemcitabine 100 mg/kg/day twice a week i.p., or the combination. Treatment was continued for 3 weeks at which point the mice were euthanized and the tumors examined. The largest tumor each from the ICG-001 alone treated group and the PBS treated group was subsequently used for secondary implantation, such that 5 NSG mice were implanted with tumor from mice previously treated with ICG-001 alone, and 5 NSG mice were implanted with tumor from mice previously treated with PBS. Three weeks after this secondary implantation gemcitabine treatment (100 mg/kg/day twice a week i.p.) was initiated. Nine days after treatment, the mice were euthanized. Therapeutic effects were judged according to tumor volume.

### 4.5. Antibodies

Primary antibodies: β-catenin from BD-Pharmingen, San Jose, CA, USA (catalog #: 610156 for immunofluorescence); β-catenin from BD-Pharmingen (catalog #: 610153 for immunoblot); survivin from Santa Cruz, Dallas, TX, USA (catalog #: sc-10811), E-cadherin from Santa Cruz (catalog #: sc-7870), CBP from Santa Cruz (catalog #: sc-369), p300 from Santa Cruz (catalog #: sc-584); K-Ras from Santa Cruz (catalog #: sc-30), Lamin A/C from Santa Cruz (catalog #: sc-7293), α-tubulin from Calbiochem, Burlington, MA, USA (catalog #: CP06), EGFR from Santa Cruz (catalog #: sc-03). Secondary antibodies for immunofluorescence were purchased from Invitrogen (Waltham, MA, USA).

### 4.6. Immunofluorescence

BxPC-3, AsPC-1, or PANC-1 human pancreatic cancer cells were plated on coverslips (250,000 cells/cm^2^) in a six-well culture plate. 24–48 h after plating, cells were washed with medium without serum and PBS and then blocked for 15 min with PBS containing 1% bovine serum albumin (BSA) (PBS-BSA). Cells were then washed with PBS, and fixed for 20 min in 4% PFA. Cells were washed with PBS, and then permeabilized for 10 min with 0.2% Triton X-100. Cells were washed with PBS, and then blocked for 15 min in PBS-BSA. Cells were washed with PBS, and then incubated for 1 h with 100 µL primary antibody against β-catenin diluted in PBS-BSA. Cells were washed with PBS, and then blocked for 15 min in PBS-BSA. Cells were washed with PBS, and then incubated for 30 min with 100 µL secondary antibody conjugated to FITC diluted in PBS-BSA.

### 4.7. TOPFlash/FOPFlash and Survivin Reporter Assays

TCF promoter-luciferase constructs TOPFlash and FOPFlash (containing eight wild-type TCF/LEF binding sites and mutant consensus sequences, respectively), as previously described [35], were used to determine the basal transcriptional activity of the β-catenin/Tcf complex in BxPC-3, AsPC-1, and PANC-1 human pancreatic cancer cells. Similarly, survivin transcription was investigated using the survivin/luciferase promoter construct (pLuc-6270) as previously described [31].

### 4.8. Co-Immunoprecipitation

We studied the effect of various treatments as indicated on CBP/β-catenin and p300/β-catenin association by co-immunoprecipitation as previously described [25]. Briefly, pancreatic cancer cells were treated with ICG-001 for 24 h, and cytoplasmic and nuclear protein fractions were extracted. From the nuclear fractions, we immunoprecipitated CBP and p300 on agarose beads. The eluents were run on standard sodium dodecyl sulfate polyacrylamide gel electrophoresis (SDS-PAGE) and transferred to a polyvinylidene difluoride (PVDF) membrane. Membranes were immunoblotted for β-catenin.

### 4.9. Lentiviral shRNA Knockdown of K-Ras

PANC-1 cells were transduced with lentiviral short hairpin RNA (shRNA) targeting *K-Ras* (Santa Cruz, catalog #: sc-35731-v), empty control (Santa Cruz, catalog #: sc-108080), or control lentivirus expressing green fluorescent protein (GFP) (Santa Cruz, catalog #: sc-108084). Briefly, cells were plated into 15-cm dishes at 5 million cells per plate. The cells were allowed to attach and grow for 48 h prior to lentiviral infection. After removal of old medium, 10 mL of fresh medium containing 5 µg/mL of polybrene was added to the cells. 2 µL of the lentiviral supernatant was added and mixed gently prior to incubating the cells overnight. Next day, the medium containing the polybrene/lentiviral supernatant was removed and fresh medium was added. After three days, cells were put under 2 µg/mL puromycin selection. Five independent clones were tested and the one showing the best knockdown of *K-Ras* by Western blot was used for further analysis.

### 4.10. Western Blot

For immunoblot analysis of nuclear and cytoplasmic extracts from cells in the shRNA experiments, extracts were prepared as previously described [31]. Otherwise, expression of protein was evaluated by standard Western blot (immunoblot) of whole protein extracts from pancreatic cancer cells treated for 48 h with various pharmacologic agents as indicated. Protein extracts were separated on SDS-PAGE, transferred to PVDF membrane and immunoblotted as previously described [25].

### 4.11. Reverse Transcription Quantitative Polymerase Chain Reaction (RT-qPCR) Analysis

Changes in the gene expression were assessed by SYBR-Green RT-qPCR. Pancreatic cancer cells were treated with various pharmacologic agents as indicated. Cells were grown in Dulbecco’s Modified Eagle’s Medium with 10% fetal bovine serum. On the day prior to treatment, 250,000 cells were seeded into 6-well plates. Cells were allowed to attach overnight. Cells were treated for 48–96 h and harvested with Trizol Reagent (Life Technologies, Waltham, MA, USA). Total RNA was extracted per manufacturer’s protocol. One microgram of total RNA was used to carry out first strand cDNA using the cDNA Synthesis Kit (Quanta, Beverly, MA, USA). RT-qPCR analysis was performed on the Bio-Rad MyiQ Single Color Real-Time PCR Detection System using PerfeCTa SYBR Green SuperMix (Quanta) and the following forward and reverse primers: Survivin—5′-AGCCCTTTCAAGGACCAC-3′ and 5′-GCACTTTCTTCGCAGTTTCC-3′; let-7a—5′-CCTGGATGTTCTCTTCACTG-3′ and 5′-GCCTGGATGCAGACTTTTCT-3′; GAPDH—5′-GGTGCTGAGTATGTCGTGGA-3′ and 5′-ACAGTCTTCTGGGTGGCAGT-3′; β-actin—5′-AGGAGCACCCCGTGCTGCTGA-3′ and 5′-CTAGAAGCATTTGCGGTGGAC-3′. High-throughput, real-time PCR studies were performed using SmartChip (WaferGen Bio-systems, Inc., Fremont, CA, USA) as per the manufacturer’s protocol.

### 4.12. Data Analysis

Numerical data were expressed as the means ± standard error of the means (SEM) unless otherwise noted. Student’s t-test was performed to assess the statistical significance between two sets of data; *p* values less than 0.05 were considered significant.

## 5. Conclusions

We report for the first time that K-Ras activation increases the CBP/β-catenin interaction in pancreatic cancer; and that ICG-001 specific antagonism of the CBP/β-catenin interaction sensitizes pancreatic cancer cells and tumors to gemcitabine treatment. These effects were associated with increases in the expression of let-7a microRNA; suppression of K-Ras and survivin; and the elimination of drug-resistant cancer stem/tumor-initiating cells.

Importantly, the combination of ICG-001 plus gemcitabine dramatically decreased the volume of tumors in our human patient-derived xenograft (PDX) model of PDAC. Moreover, the toxicity of gemcitabine in this model, manifested by reduced body weight in mice treated with gemcitabine alone, was ameliorated in the ICG-001 plus gemcitabine combination group. Although ICG-001 alone did not inhibit the growth of the PDX tumor upon initial transplantation, in secondary implantation experiments without any further treatment, the ICG-001 treated tumors failed to grow. These results further demonstrate the ability of ICG-001 to safely and selectively target the PDAC cancer stem/tumor initiating cell population in vivo. Although further clinical investigations will be required to optimize this therapeutic regimen, we believe this approach represents a truly novel and broadly applicable differentiation strategy for PDAC and cancer more generally.

## Supplementary Materials

The following are available online at http://www.mdpi.com/2072-6694/10/4/95/s1, Figure S1: Total levels of CBP, p300, and β-catenin in PANC-1 cells treated with ICG-001 for 24 h. Figure S2: Total levels of CBP, p300, and β-catenin in PANC-1 cells treated with ICG-001 or PD98059 for 16 h. Table S1: AsPC-1 ICG-001 vs. DMSO 96 h fold changes in gene expression.
