# Supplementary material for: Differentiation Therapy Targeting the β-Catenin/CBP Interaction in Pancreatic Cancer
Source: Cancers (Basel). 2018 Mar 29;10(4):95. doi: 10.3390/cancers10040095 (PMC5923350; doi:10.3390/cancers10040095)
Supplement: Supplementary file 1 [file cancers-10-00095-s001.zip › cancers-277092-supplemantary Materials/Figures S1 and S2.pdf]

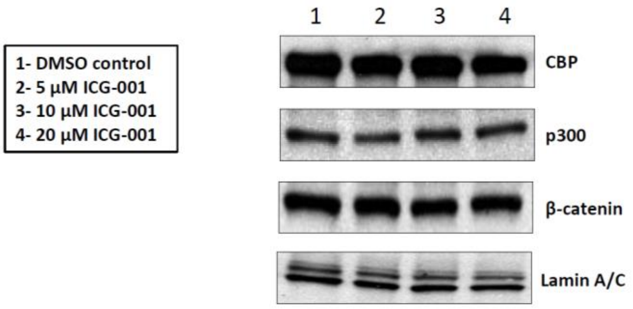

Figure S1. Total levels of CBP, p300, and β-catenin in PANC-1 cells treated with ICG-001 for 24 h. Treatment with ICG-001 does not change total nuclear β-catenin, CBP, and p300 levels.

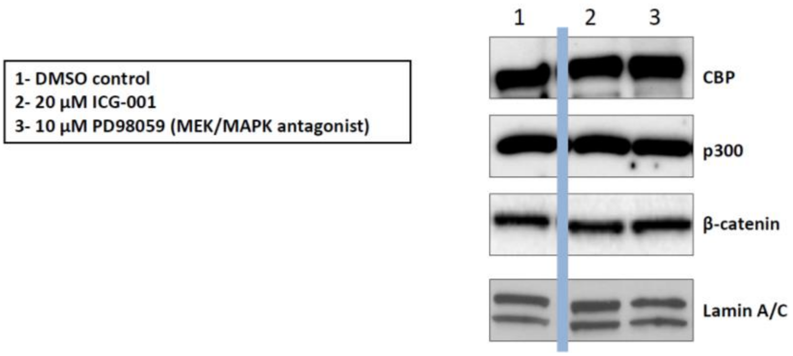

Figure S2. Total levels of CBP, p300, and β-catenin in PANC-1 cells treated with ICG-001 or PD98059 for 16 h. Treatment with ICG-001 or PD98059 does not change total nuclear β-catenin, CBP, and p300 levels. Blue dividing line indicates splice junction.
